# Camphor for Ether Collapse

**Published:** 1896-05

**Authors:** 


### Camphor for Ether Collapse.

Schilling (*Munchener Med. Wochen.*), affirms that hypodermic injections of camphor, in larger doses than the text-books advise, are beneficial in ether collapse. Half-grain doses are very effective, but the results obtained from one-grain doses are extremely gratifying. The solution should be one part camphor to ten parts oil. As the camphor is eliminated within two hours it has no cumulative effect.—*N. Y. Med. Record.*

